# Current methods for analyzing time-series patient-generated health data to assess treatment response: a scoping review

**DOI:** 10.1093/jamia/ocag027

**Published:** 2026-03-11

**Authors:** Michelo Banda, Sian Bladon, Mariam Al-Attar, Roberto Cahuantzi, David A Jenkins, William G Dixon, Sabine N van der Veer

**Affiliations:** Division of Informatics, Imaging and Data Science, Manchester Academic Health Science Centre, The University of Manchester, Manchester, M13 9GB, United Kingdom; Department of Pharmacy, School of Health Sciences, University of Zambia, Lusaka, 10101, Zambia; Division of Informatics, Imaging and Data Science, Manchester Academic Health Science Centre, The University of Manchester, Manchester, M13 9GB, United Kingdom; Division of Informatics, Imaging and Data Science, Manchester Academic Health Science Centre, The University of Manchester, Manchester, M13 9GB, United Kingdom; Division of Informatics, Imaging and Data Science, Manchester Academic Health Science Centre, The University of Manchester, Manchester, M13 9GB, United Kingdom; Division of Informatics, Imaging and Data Science, Manchester Academic Health Science Centre, The University of Manchester, Manchester, M13 9GB, United Kingdom; Division of Informatics, Imaging and Data Science, Manchester Academic Health Science Centre, The University of Manchester, Manchester, M13 9GB, United Kingdom; NIHR Manchester Biomedical Research Centre, Manchester University NHS Foundation Trust, Manchester Academic Health Science Centre, Manchester, M13 9WL, United Kingdom; Division of Informatics, Imaging and Data Science, Manchester Academic Health Science Centre, The University of Manchester, Manchester, M13 9GB, United Kingdom

**Keywords:** patient-generated health data, treatment response

## Abstract

**Objectives:**

We aimed to identify and map recent studies using high-frequency, time-series electronic patient-generated health data (ePGHD) to assess treatment response; characterize ePGHD types and collection methods; summarize ePGHD-based definitions of treatment response; and describe analytical approaches used.

**Materials and Methods:**

We systematically searched 4 databases for articles published between January 2022 and June 2024, supplemented by a forward citation search until June 2025. Peer-reviewed studies were eligible if ePGHD were collected outside clinical settings, and either reported at least weekly (ie, if actively reported by participants) or summarized discretely (eg, daily) if passively collected via wearables/sensors. We screened articles for eligibility independently in duplicate and synthesized extracted data descriptively.

**Results:**

Our search yielded 4030 articles, of which we included 186. Most studies collected ePGHD using mobile applications or webforms (*n* = 133) over 4-12 weeks (*n* = 67). Prior to analysis, 132 studies excluded portions or condensed ePGHD into one or more summaries. Among 172 studies estimating treatment response, 98 applied longitudinal methods (eg, mixed-effects models) that accounted for repeated measures while capturing within- and between-subject variations, whereas 74 used cross-sectional approaches. Of 18 prediction modeling studies, 16 employed machine learning techniques, with only 4 explicitly modeling repeated measures. Five studies identified clusters of response trajectories generally without incorporating temporal dependencies (eg, using K-means).

**Discussion and Conclusion:**

Many studies in this review did not fully leverage the high-frequency, longitudinal nature of ePGHD. Future research should adopt more appropriate and readily available analytic methods to maximize the potential of time-series ePGHD for generating insights into treatment response.

## Introduction

The assessment of treatment response is a key aspect of disease management that draws on evidence from clinical studies to support and guide clinical decision-making.^1^ Traditionally, outcomes are evaluated using clinical measures alongside patient-reported symptoms and behaviors recalled over months between clinic appointments.^2^^,^^3^ Increasingly, there is recognition that capturing changes in patients’ clinical states between appointments is important, as disease fluctuations can influence treatment response assessment and subsequent decisions.^3^^,^^4^

Digital technologies, such as smartphones and wearables, have enabled patients to generate electronic patient-generated health data (ePGHD) at home in real-time. Electronic patient-generated health data include: (1) patient experience and opinion such as symptoms, quality of life, and adverse effects of treatments; (2) behavior and exposure data including diet, treatment adherence, acute medication use, sleep, and physical activity; and (3) physiological data such as blood glucose, blood pressure, and electrocardiogram readings.^5^ Unlike clinical data, ePGHD are captured by patients or carers without involvement of a health-care professional and shared at the patient’s discretion.^6^

Frequent ePGHD capture provides real-time measurement of the “ups and downs” of changing disease severity through time, including the opportunity of assessing treatment response as it happens. It also reduces recall bias associated with infrequent self-reporting, thereby enhancing data quality and reliability.^3^^,^^7^ Additionally, ePGHD are valuable in contexts where laboratory tests or imaging may be unavailable, such as in chronic pain.^8^

The value of ePGHD often lies in frequent collection (eg, at least weekly), but analyzing high-frequency time-series data requires additional methodological considerations compared with infrequent measurements, such as accounting for correlations between repeated observations.^9^ While prior reviews examined the use of ePGHD to assess postsurgical outcomes,^10^ or cancer treatment response,^11^ they did not describe how ePGHD were handled or analyzed. This scoping review addresses this gap by summarizing data handling and analytical approaches used by recent research to assess treatment response, focusing on studies in populations with a health condition who reported ePGHD independently from home at least weekly. Specifically, we aimed to: (1) identify and characterize studies that used high-frequency time-series ePGHD to assess treatment response, (2) describe what ePGHD types were collected and how, as well as ePGHD-based definitions of response, and (3) describe ePGHD handling and analysis methods.

## Methods

We conducted a scoping review in line with the exploratory nature of our effort, aiming to identify, map and examine data handling and analysis methods currently being applied for high-frequency time-series ePGHD to assess treatment response.^12^ We adhered to methodological guidance by the Joanna Briggs Institute,^13^ and reporting guidance from the Preferred Reporting Items for Systematic Reviews and Meta-Analyses extension for Scoping Reviews (PRISMA-ScR) checklist.^14^ The protocol was registered to Open Science Framework at https://osf.io/ec7u9.

### Search strategy

We conducted a systematic electronic search of Ovid Medline, Ovid Embase, Web of Science, and ACM digital library for eligible studies on June 15, 2024, which we supplemented with a manual forward search conducted 1 year later (June 9-14, 2025).

### Search terms

We developed a search syntax to cover the concepts of ePGHD (including terms for digital devices commonly used for ePGHD collection) and treatment response (including terms for common ePGHD-based outcomes) building on and refining terms from Pyper et al.,^15^ in collaboration with an experienced librarian. Searches were conducted across the 4 databases, with full strategies provided in Tables S1-S4.

### Study selection

We included English-language, peer-reviewed studies, full conference papers, and published protocols from January 2022 to June 2024, focusing on current methods for assessing treatment response using ePGHD. Gray literature, reviews, commentaries, letters, opinions, and conference abstracts were excluded. Studies using wearable or sensor-derived ePGHD were included only if data were summarized to discrete intervals (eg, once or twice daily); studies analyzing exclusively continuous, high-frequency data streams were excluded. A forward citation search (June 9-14, 2025) identified additional studies. Eligibility criteria are summarized in Table 1 and detailed in Table S5.

**Table 1.** Eligibility criteria.

Item	Definition
Population	People of any age with any health condition undergoing any type of treatment
Concept	i) High-frequency ePGHD - Any health-related data reported electronically by patients and/or their proxies at least once weekly independently and without researcher or clinician assistance, and relevant to disease management ii) Treatment response assessment - Evaluation of changes in health status and related treatment decisions following an intervention, including efficacy, effectiveness, and adverse events
Context	Data collected outside clinical, hospice, or assisted-living settings
Study type	Longitudinal studies

Abbreviation: ePGHD, electronic patient-generated health data.

Search results from the database searches were imported into Covidence systematic review software (Veritas Health Innovation, Melbourne, Australia), and deduplicated. Screening occurred in 2 stages (title/abstract and full text). One reviewer (M.B.) screened all records, with a second reviewer (S.B., M.A., or R.C.) independently screening in duplicate; disagreements were resolved by consensus. Data extraction was conducted by M.B.

### Data extraction and synthesis

To address objective 1, we extracted study characteristics (eg, year, design, health condition, sample size). For objective 2, we collected details on ePGHD type, reporter, reporting method, frequency, duration, and ePGHD definitions of treatment response. For objective 3, we extracted information on missing data reporting, preprocessing (eg, summarization, thresholds, imputation), and analytical methods; capturing only the most appropriate for synthesis when multiple methods were used (see Table S6). Results were synthesized using frequencies and percentages and presented in tables and visualizations.

## Results

### Study search and selection

**Figure 1.**
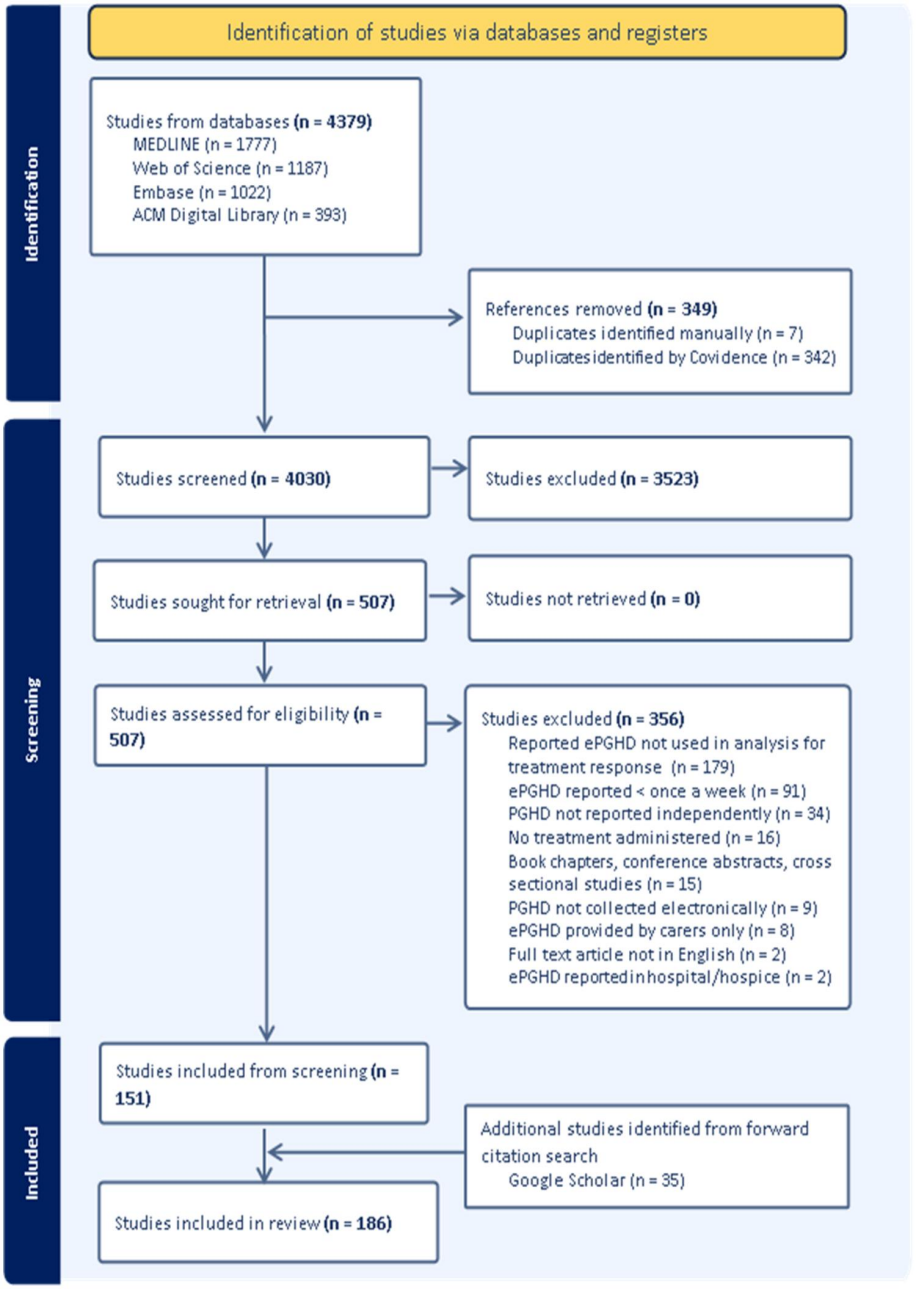
PRISMA flow chart of our search and selection process. The systematic search from 2022 to June 2024 identified 151 studies for inclusion. Forward citation search of the 151 included studies from June 2024 to June 2025 led to inclusion of an additional 35 studies. Abbreviation: PRISMA, Preferred Reporting Items for Systematic Reviews and Meta-Analyses.

Figure 1 shows our process of searching for and selecting relevant studies published since 2022. From 4030 unique records identified in the June 2024 database search, 151 studies met inclusion criteria. The most common reasons for full-text exclusion were failure to incorporate ePGHD into treatment response analyses (*n* = 179) and reporting ePGHD less than weekly (*n* = 91). A forward citation search in June 2025 identified 35 additional studies, yielding 186 included studies in total.

### Study characteristics

Table 2 shows that many studies were conducted in North America (*n* = 77, 41%), designed as randomized controlled trials (*n* = 84, 45%), enrolled fewer than 100 participants (*n* = 79, 42%), and focused on adults (*n* = 171, 92%). Over one-quarter studied neurological conditions (*n* = 53, 28%), 40 of which specifically addressed migraine and sleep disorders. Across the 186 studies, drug treatment was the most frequently employed intervention (*n* = 84, 45%). Additional study-level information is provided in File S2.

**Table 2.** Characteristics of included studies (total *n*=186).

Study characteristic	*n* (%)
Publication year	
2022	49 (26)
2023	63 (34)
2024	61 (33)
2025	13 (7)
Publication type	
Journal article	155 (83)
Study protocol	25 (13)
Conference paper	6 (3)
Study design	
Randomized controlled trial	84 (45)
Observational study	52 (28)
Other interventional study, for example, prepost study	50 (27)
Geographic location	
North America	77 (41)
Europe	49 (26)
Asia	21 (11)
South America	2 (1)
Australia	1 (1)
Multicontinental	32 (17)
Not reported	4 (2)
Health condition	
Neurology	53 (28)
Oncology	23 (12)
Mental health and addictions	21 (11)
Cardiovascular	19 (10)
Metabolism and endocrinology	15 (8)
Musculoskeletal	15 (8)
Respiratory/pulmonary	9 (5)
Gastroenterology	8 (4)
Women’s health	8 (4)
Pain	5 (3)
Dermatology	3 (2)
Rheumatology	3 (2)
Hematology	2 (1)
Other (eg, amputation caused by various reasons)	2 (1)
Sample size	
<100	79 (42)
100-500	73 (39)
>500	34 (18)
Population type^a^	
Adults (≥18)	171 (92)
Children (<18)	12 (6)
Not reported/unclear	3 (2)
Intervention	
Drug treatment	84 (45)
Other nonpharmacological intervention (eg, app-based adaptive exercise programs, in-person or tele-rehabilitation programs, cognitive behavioral therapy)	64 (34)
Procedure (eg, surgery)	28 (15)
Drug treatment + other nonpharmacological intervention	4 (2)
Device (eg, pacemaker)	3 (2)
Drug treatment + procedure	3 (2)

a Some studies that included overlapping age categories were assigned based on mean/median age.

### ePGHD characteristics and treatment response definitions

As shown in Table 3, about one-third of studies (*n* = 64, 34%) solely collected ePGHD on participants’ experiences and opinions, with fewer collecting behavior and exposure data (*n* = 35, 19%) and physiological measures (*n* = 19, 10%). However, studies often collected multiple types of ePGHD in different combinations (*n* = 68, 37%), for example, pain experiences and physical activity behavior,^16^ or of dietary behavior, treatment adherence, and blood glucose measures.^17^

**Table 3.** ePGHD characteristics and treatment response definitions.

ePGHD characteristics	*n* (%)
ePGHD type	
Patient experience and opinion, for example, symptoms, adverse effects	64 (34)
Behavior and exposure, for example, physical activity, sleep	35 (19)
Physiological data, for example, blood pressure, lung function measures	19 (10)
Multiple types	68 (37)
ePGHD reporter	
Patient	177 (95)
Proxy (parent or carer)	2 (1)
Patient and/or proxy	7 (4)
ePGHD reporting type	
Active only	114 (61)
Passive only	42 (23)
Both active and passive	30 (16)
ePGHD reporting method	
Mobile app	78 (42)
Web-based form, for example, weblink	55 (30)
Wearable/sensor/implant	19 (10)
Interactive voice response system/short messaging service	3(2)
Connected device (camera, spirometer)	2(1)
Multiple methods	29 (16)
ePGHD reporting frequency	
At least once daily	143 (77)
At least once weekly	23 (12)
Multiple frequencies	20 (11)
ePGHD reporting durations^a^	
≤1 week	9 (5)
>1-4 weeks	24(13)
>4-12 weeks	67 (36)
>12-26 weeks	46 (25)
>26 weeks	40 (22)
ePGHD-based treatment response definition	
None provided	143 (77)
Reduction in frequency and/or severity of symptoms	26 (15)
Increase or reduction in physiological measures, for example, blood pressure, blood glucose	11 (6)
Increase or reduction in behavioral activity, for example, increased physical activity or reduced intake of acute medication	5 (3)

Abbreviation: ePGHD, electronic patient-generated health data.

a Longest reporting period used to assign studies with participant ePGHD spanning different categories.

In most studies, only patients themselves reported ePGHD (*n* = 177, 95%), mainly through active input (*n* = 114, 61%) via mobile apps (*n* = 78, 42%) or web-based forms (*n* = 55, 30%). Most studies required daily reporting (*n* = 143, 77%) typically over 4-12 weeks (*n* = 67, 36%). For example, one migraine study collected daily app-based reports for 12 weeks.^18^ Other studies had multiple reporting frequencies (*n* = 20, 11%), such as one where participants reported socket comfort levels daily and side effects weekly following a prosthetic fitting.^19^

Only 42 of 186 studies (23%) explicitly provided definitions of treatment response. Of these, 16 were from the field of neurology (38%), followed by metabolism and endocrinology (*n* = 8, 19%). Definitions typically focused on reductions in symptom frequency and/or severity (*n* = 26) and were operationalized as point changes, percentage changes, or clinical thresholds. To illustrate, when investigating the effect of medication in preventing blood pressure spikes, one study defined response based on a clinical threshold of ≥140/90 mmHg.^20^ Another study defined treatment response as a 50% reduction in the number of migraines reported per month.^21^ Studies that did not provide explicit definitions of treatment response inferred this solely from statistical differences.

### Data handling and analysis methods

#### Missing ePGHD

Of the 186 studies in this review, 25 were protocols and 161 presented results. Of the latter, 114 (71%) reported having missing data, with 51 setting ePGHD completeness thresholds for including participants in subsequent analysis. Thresholds ranged from “at least one ePGHD-based observation” (eg, literature^22^^,^^23^) to complete reporting (ie, 100% data completeness, eg, literature^24^^,^^25^). To illustrate, one study included for analysis only participants who reported sleep data daily, excluding those without complete sets from analysis.^25^

Of 161 studies with results, 56 (35%) reported the pattern of ePGHD completion. Of these, 12 had a declining reporting pattern over time, while 44 had stable reporting patterns. For instance, in one asthma study with weekly symptom reporting, 373 participants completed baseline questionnaires, but reporting declined sharply to 19 participants at 3 months and 11 at 6 months.^26^ In contrast, another study had more stable reporting, with over 90% of participants continuing to submit weight data at both 3- and 6-month follow-up.^27^

Only a small proportion (*n* = 25, 13%) of the 186 studies imputed/planned to impute missing ePGHD, using methods such as prorating,^28–34^ linear interpolation,^35–38^ multiple imputation,^19^^,^^39–42^ mean imputation,^43^ and last-observation-carried-forward.^44^^,^^45^ Nine studies analyzed observed ePGHD alongside sensitivity analyses where missing ePGHD were imputed, comparing estimates from both analyses. Imputation methods included Markov chain Monte Carlo,^46^ pattern mixture model,^46^^,^^47^ and predictive mean matching.^48^

Figure S1 provides a detailed breakdown of how studies reported and handled missing data (see File S1). More study-level information can be found in File S2.

#### Preprocessing of ePGHD

**Figure 2.**
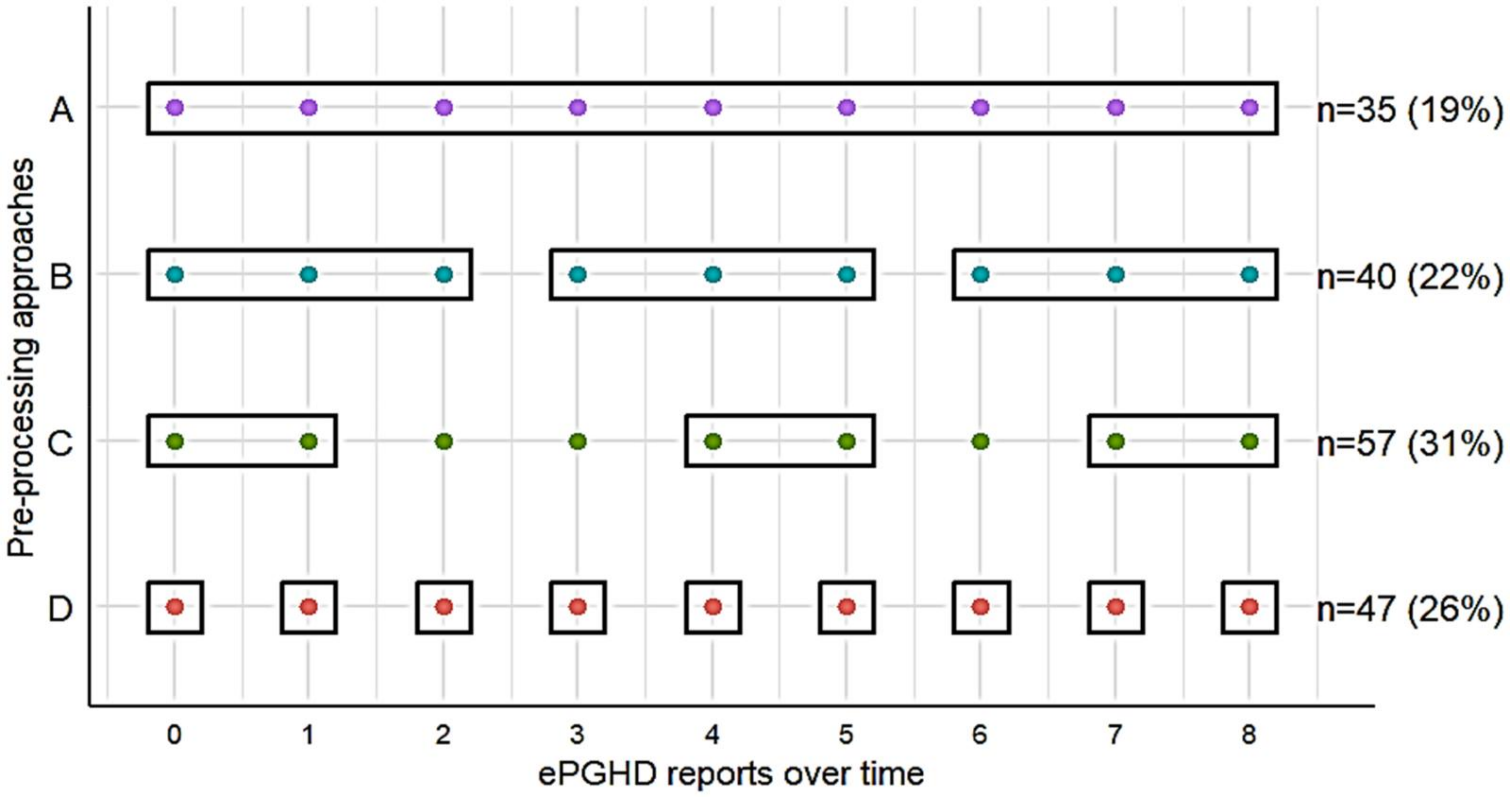
Approaches to preprocessing of ePGHD. Each dot represents an observation, while rectangles show how observations were summarized across time in rows A-C. Dots not surrounded by rectangles in row C show observations were excluded from analyses. Squares around dots in row D show that each observation was analyzed as reported. Seven studies did not provide information about preprocessing approaches. Abbreviation: ePGHD, electronic patient-generated health data.

Figure 2 shows that studies used 1 of the 4 different approaches (A-D) to preprocess ePGHD, ranging from summarizing ePGHD into a single summary (A, eg, literature^49^^,^^50^; *n* = 35, 19%) or across multiple discrete contiguous time periods (B, eg, literature^51^^,^^52^; *n* = 40, 22%) to using selected data points and disregarding those remaining (approach C; eg, literature^53^^,^^54^; *n* = 57, 31%) or using all data points individually (D, eg, literature^55^^,^^56^; *n* = 47, 26%).

As an example of approach A, one study summarized 3 months of participants’ daily pain intensity scores into single mean values and compared these between the intervention and control group to evaluate the effect of app-delivered relaxation exercises.^57^ One study that used approach B asked cancer patients to report their fatigue levels weekly for 12 weeks following a digital intervention, then condensed these to 3 contiguous 4-week mean scores before analysis.^58^ To investigate the effect of drug treatment on abdominal pain and bowel urgency, one study asked participants to report daily scores, using only their 3-day averages that preceded clinical visits at weeks 2, 4, 8, 12, 20, 28, 36, and 52 for analysis,^39^ thus processing data using approach C. Another study that used this approach asked parents of children with sleep problems to report their daily sleep metrics for at least 8 weeks, but only used baseline and end of follow-up measures for analysis.^59^ A study using approach D measured the efficacy of drug treatment in delaying adverse symptoms from sun exposure by including each daily report as an individual measurement in the analysis.^46^

#### ePGHD analysis methods

**Figure 3.**
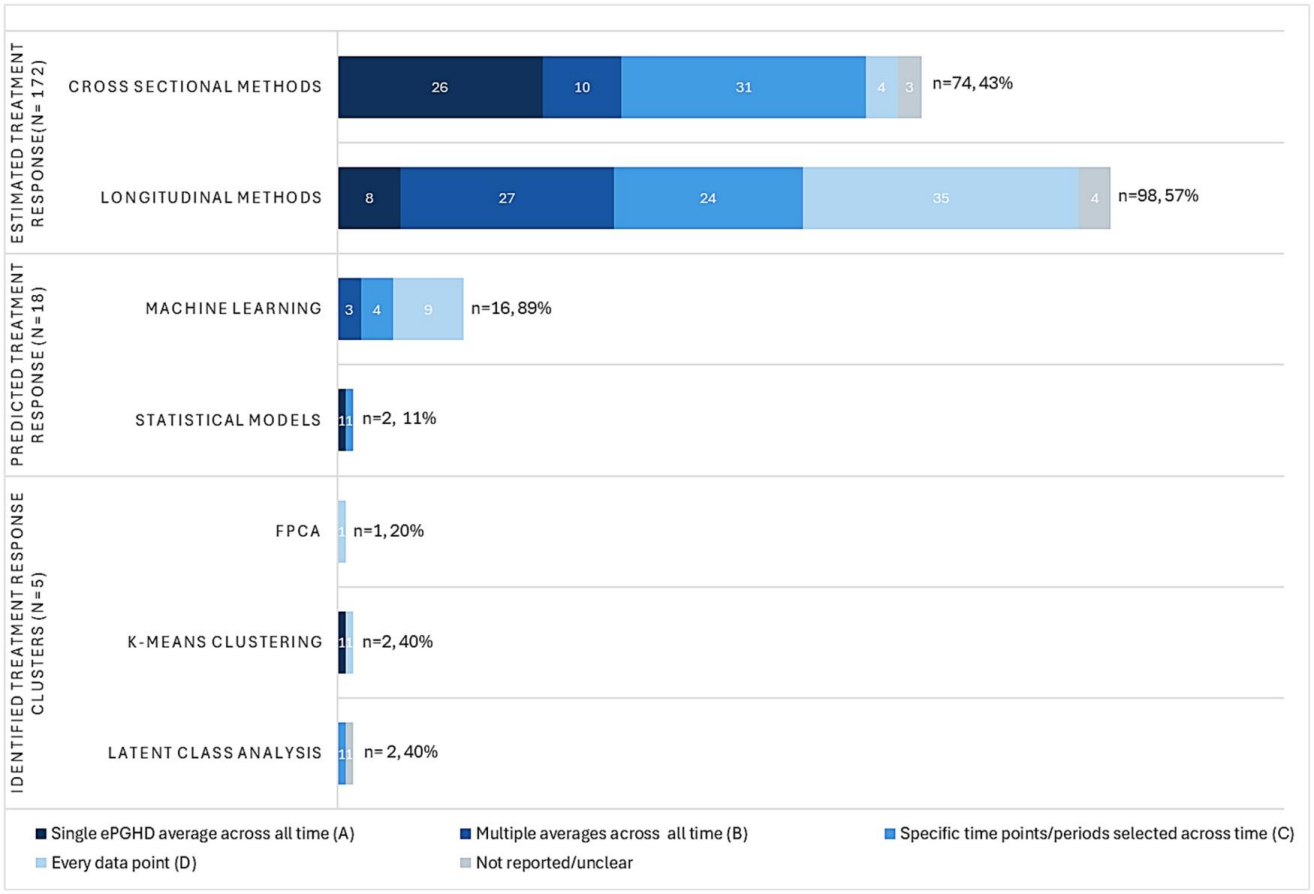
Methods and preprocessing approaches used. Color coding refers to preprocessing approaches from Figure 2. For studies that estimated treatment response, we grouped those based on their ability to analyze longitudinal data. Cross-sectional methods included descriptive summaries, Pearsons or Spearman’s correlation tests, independent *t*-test, Wilcoxon signed-rank test, Kruskal-Wallis H test, Chi-square test, Fischer’s exact test, Cochran-Mantel-Haenszel test, Kaplan-Meier curves, Cox proportional hazards, and general linear models. Longitudinal methods included paired *t*-test, repeated measures ANOVA, mixed-effects models, generalized estimating equations, structural equation modeling and hierarchical Bayesian models. Studies predicting treatment response were categorized according to groupings commonly found in the literature (ie, machine learning and statistical models). Statistical models included logistic regression, while machine learning models were K-nearest neighbors, decision trees, support vector machine, ensembles (random forest, gradient boost), neural networks, elastic net regression, and kernel approximation. Since studies that clustered ePGHD trajectories were few, actual methods are shown. Abbreviations: ePGHD, electronic patient-generated health data; FPCA, functional principal component analysis.

We synthesized ePGHD analysis methods by grouping studies into 3 objectives, noting that some addressed multiple objectives. These included studies estimating treatment response or its association with ePGHD, studies using ePGHD to predict treatment response, and studies identifying clusters of ePGHD trajectories representing distinct response subgroups. Figure 3 presents the number of studies in each group, further subdivided based on their analysis methods and abovementioned preprocessing approaches (see Figure 2).

##### Estimating treatment response

Figure 3 shows that among the 172 studies estimating treatment response, most applied longitudinal methods (*n* = 98, 57%) rather than cross-sectional methods (*n* = 74, 43%). In addition, most studies using longitudinal methods analyzed every data point (*n* = 35), which corresponds to approach D in Figure 2, followed by use of multiple summaries (*n* = 27), corresponding to approach B. The most employed longitudinal method were mixed-effects models (*n* = 65, 66%), followed by paired *t*-tests (*n* = 14, 14%) and repeated measures ANOVA (*n* = 12, 12%) (see File S2 for study-level information). To illustrate how longitudinal methods used the 2 preprocessing approaches, one study which evaluated the efficacy of drug treatment for hypotension in individuals with spinal cord injury used every self-reported blood pressure reading collected multiple times per day in a mixed-effects model, accounting for repeated measures and their correlation by including participant as a random effect.^55^ Another study that evaluated the impact of an intervention on daily physical activity in people with heart failure aggregated the daily scores into weekly measures before using repeated measures ANOVA to assess both within- and between-group differences over time.^60^

Of the 74 studies using cross-sectional methods, over a third (*n* = 27, 36%) used general linear models for ePGHD analysis, while over a quarter only applied descriptive statistics (*n* = 21, 28%). Furthermore, as shown in Figure 3, most studies using cross-sectional methods used specific data points (such as approach C in Figure 2), while many others condensed all reported ePGHD into a single measure (approach A in Figure 2). To demonstrate the use of these methods and approaches, one study evaluating a digitally delivered weight management program summed participants’ time spent on the app over the 12-week intervention and used a linear regression model to examine how this variable was related to weight loss.^61^ Another study that asked people with migraine to record migraine-free status multiple times within a 2-h period after drug treatment reported only the proportion of participants who were migraine-free at the 2-h mark.^62^

##### Predicting treatment response

Eighteen studies developed prediction models that utilized ePGHD to forecast treatment response. Treatment response outcomes were often based on ground truth and included validated patient-reported outcomes such as quality of life measures,^63^ clinical measures such as HbA1c,^64^ or other ePGHD endpoints such as substance abuse.^65^ As depicted in Figure 3, most of the studies (*n* = 16, 89%) used machine learning for prediction tasks, which included, for example, random forests, gradient boosting, elastic net regression, and support vector machines.

Most studies that employed machine learning models for prediction tasks analyzed all reported ePGHD (approach D in Figure 2; *n* = 9). For instance, one study that collected ecological momentary assessments and physical activity metrics before surgery used all scores to predict postoperative recovery.^66^ In contrast, one study that used a statistical model (ie, logistic regression) to predict clinical escalation of therapy in patients with ulcerative colitis who reported their symptoms daily or once weekly for 6 months only used ePGHD reported within 5 days of their clinical visit for prediction tasks (approach C in Figure 2).^41^

Only 4 of 18 (24%) studies predicting treatment outcomes explicitly accounted for repeated measures using approaches such as random effects,^65^ gated recurrent units and sequence-to-sequence modeling,^67^ or multilevel dynamic structural equation modeling embedded within machine learning models.^66^^,^^68^

##### Clustering treatment response

Figure 3 shows that only 5 studies used ePGHD to identify clusters of treatment response. Only one study explicitly accounted for between-subject variation when identifying clusters using functional principal component analysis. This model was applied to daily ecological momentary assessments and physical activity measures, for which different postoperative recovery trajectories were found.^56^

Studies that used K-means and latent class analysis did not describe any techniques for accounting for within- and between-subject variations.^20^^,^^68^^,^^69^

## Discussion

### Summary of findings

This review included 186 studies published since 2022 assessing treatment response using ePGHD. Many were conducted in North America, focused on neurology, and had ≤500 participants. Data were typically self-reported by patients through apps or webforms over 4-12 weeks. Few studies defined treatment response, and many did not provide information on missing data or reporting patterns over time. While some studies applied data completeness thresholds for including participants in analyses, most did not. Data preprocessing often involved generating one or more summary measures from the full dataset, and in a third of cases, excluding portions of available ePGHD. While some studies accounted for repeated measures and captured within- and between-person variation for estimation, clustering, or prediction tasks, other studies relied on analyses that treated observations as independent and ignored these correlations.

### Relation to other studies

Although all studies collected repeated ePGHD from the same participants, many used methods treating observations as independent, regardless of whether estimating, predicting, or clustering treatment response. This finding is in keeping with similar reviews in oncology^70^ and physical activity.^71^

Most studies preprocessed ePGHD by summarizing data over time, excluding portions of available data, or applying completeness thresholds. This is in line with a review of data preprocessing techniques for passively collected ePGHD in psychosis and schizophrenia.^72^ In addition, while many studies in our review did not include information on missing data, an even larger number did not describe participant reporting patterns. This finding aligns with other reviews of ePGHD studies, which observed that it was seldom specified whether data were missing,^70^^,^^72^ or what reporting patterns were.^73^

### Study limitations

This review has some limitations. Our search terms may not have captured studies that used different terminology in their titles or abstracts. For example, research using names of mobile apps as opposed to the term “mobile app” may have been missed, potentially leading to an underestimation of published literature. Although limiting our review to studies published from 2022 onward was in line with the study’s aim, broadening the timeframe would have enabled a more comprehensive picture of methodological trends and assessment of whether practices have been improving over time. This may be the focus of a future review.

Rather than updating our initial search, we used forward citation searching to identify recently published studies. The reliance of this approach on variable and potentially skewed citation practices means we may have missed relevant, more recent work that was not explicitly building on published research.

This review did not extract or synthesize information on categorization of continuous ePGHD or handling of extreme values. Such practices can influence the precision and robustness of analyses using high-frequency ePGHD. Continuous predictors are often most informative when analyzed on their native scale, with flexible approaches (eg, fractional polynomials or restricted cubic splines) used to accommodate nonlinearity,^74^^,^^75^ while extreme values may be addressed using robust modeling, sensitivity analyses, or clinically guided filtering.^76^ A future review could examine how frequently and transparently such practices are reported and implemented.

### Implications for future research

#### Defining treatment response to enable clinical translation

Fewer than one-quarter of studies explicitly defined treatment response, with most inferring effects solely from statistical differences. Statistical significance alone does not establish clinical relevance, as it does not indicate whether observed changes represent meaningful improvement for patients or clinicians in the absence of predefined clinical thresholds.^77–79^ This also limits comparability across studies, because without explicit response criteria, it becomes unclear whether studies are measuring conceptually similar treatment effects, hindering evidence synthesis and generalization.^80^ Future studies should adopt explicit, clinically meaningful response definitions, such as minimal clinically important differences or responder thresholds, to improve interpretability and support application in practice.

#### Adopt more robust longitudinal methods to account for correlation between repeated ePGHD measures

Many studies estimating treatment response in our review applied cross-sectional methods such as general linear models or descriptive summaries analyzing ePGHD from selected time points. This approach allows simplified estimation of treatment response at specific times, for example, the proportion of participants reaching a threshold or showing a statistically significant difference. However, these methods treat ePGHD observations at each time point independent of participants observations taken at other times, therefore ignoring correlations between measures. As a result, estimates can be less precise and may fail to fully capture within-subject changes over time as well as between-subject differences.^81^^,^^82^

Most studies in our review that employed longitudinal methods used mixed-effects models to estimate treatment response. These models are well suited to longitudinal data because they account for correlations between repeated measures, accommodate missing observations, use all available data, and capture individual differences in baseline levels and rates of change through random effects.^82^ However, some included studies applied more limited longitudinal approaches, such as paired *t*-tests and repeated measures ANOVA, which also account for correlations between repeated measures but with important constraints. Paired *t*-tests use only 2 time points and discard additional observations, preventing characterization of full within-person trajectories.^81^^,^^83^ Repeated measures ANOVA can model change over time but typically requires complete data across all time points, leading to the exclusion of participants with missing ePGHD and inefficient use of available information.^81^

The clustering methods used in the included studies did not leverage approaches capable of modeling both within- and between-subject variations, such as growth mixture models,^84^ but instead relied on methods such as K-means and functional principal component analysis that do not fully account for the longitudinal structure of the data.^82^ Similarly, many machine learning approaches for prediction can account for repeated measures and within- and between-subject variations through random effects or specialized longitudinal architectures (eg, recurrent neural networks, mixed-effects random forests)^85^; however, most prediction modeling studies in this review did not explicitly implement these features. Together, these findings suggest that across estimation, clustering, and prediction tasks, the longitudinal structure of ePGHD remains underexploited in current practice. Greater adoption of robust longitudinal methods is therefore needed to more fully realize the analytic potential of high-frequency ePGHD.

#### Leverage all available ePGHD to enhance precision and optimize participant burden

Many studies condensed ePGHD into single or multiple summaries, in some cases excluding portions of available data. While summarizing ePGHD can be appropriate in certain contexts, such as for tracking changes in migraine frequency between periods, it is suboptimal when such aggregation obscures nuanced within-period fluctuations, such as daily pain variability. Similarly, analyzing only selected portions of ePGHD also loses this detail. Summaries and data exclusions reduce the effective number of observations per participant, and thus decrease the accuracy of estimates and inflate standard errors.^86^ Consequently, studies using these approaches did not fully leverage the opportunity for more precise estimates that high-frequency time-series data provides. We therefore recommend future research to use all reported ePGHD to capture variations in response and improve estimate precision. Alternatively, researchers may consider reducing the frequency of ePGHD collection, especially in conditions without much fluctuation in health state, or when they plan to only utilize measures reported at specific times (eg, at baseline and follow-up in a comparative effectiveness trial). Requesting high-frequency reporting without full utilization in analyses imposes an undue burden on participants without added analytical value, and potentially reduces participant completion rates, which can lead to higher levels of missingness.^87^

#### Improve reporting of missing ePGHD

While reporting the presence of missing ePGHD is important, describing reporting patterns adds further value by supporting transparent interpretation of study findings. Reporting patterns may reflect patient engagement and underlying health status, providing important context for interpreting analyses when missing data may be informative.^88^^,^^89^ For example, weekend nonreporting for pain severity may occur for reasons unrelated to symptom severity but could also be informative if participants are unable to submit reports due to their health status, highlighting the need for cautious interpretation.^90^ The STROBE-CREMAS checklist for observational studies using ecological momentary assessment recommends that researchers detail missing data and reporting patterns,^73^ while the CONSORT-PRO checklist for randomized controlled trials provides guidance on reporting missing data.^91^ We recommend that studies more consistently follow these standards. Although CONSORT-PRO item 13a recommends that, “The number of participants reporting PRO data at baseline and at subsequent time points should be made transparent,” the explanation for this item and exemplar flow chart only require detailing of participant numbers at randomization, follow-up, and analysis, without necessitating capture of the number of participants submitting data at each assessment during follow-up.^91^ This makes it difficult to assess how reporting changed over time. We thus recommend extending guidance to encourage reporting of participant numbers and ePGHD availability over the entire reporting period. This can be achieved through simple time-series plots or tables (eg, literature^92–95^) that may reveal missing data trends, clarify assumptions of missingness, and improve the interpretation and validity of findings.^96^^,^^97^

## Conclusion

Since 2022, many studies have used ePGHD to assess treatment response across a range of clinical areas, with participants actively contributing data for over a month. However, data were sometimes condensed or excluded, and missingness was common but not always reported. Many studies used methods not suited to time-series data, since they incorrectly assumed measures were independent. Future research should make better use of existing, more robust approaches that explicitly account for repeated measures and within- and between-subject variabilities. Optimizing how ePGHD are analyzed can unlock their full potential to provide a clearer understanding of treatment response over time and strengthen the evidence base for clinical decision-making.

## Supplementary Material

ocag027_Supplementary_Data

## Data Availability

All data analyzed during this study are provided in Files S1 and S2.
