# Supplementary material for: Current methods for analyzing time-series patient-generated health data to assess treatment response: a scoping review
Source: J Am Med Inform Assoc. 2026 Mar 11;33(5):1065–76. doi: 10.1093/jamia/ocag027 (PMC13127660; doi:10.1093/jamia/ocag027)
Supplement: ocag027_Supplementary_Data [file ocag027_supplementary_data.zip › Supplementary file 1.docx]

Supplementary file for *Current methods for analysing time-series patient-generated health data to understand treatment response: a scoping review*

Figure S-1 below shows that studies with missing data that set thresholds for inclusion in treatment response assessments mainly excluded participants whose data fell below a specified threshold from analysis(n=39). Of these, 33 analysed the remaining participants data as it was reported, while 5 imputed any missing data before analysis. However, some studies with missing data that set thresholds still analysed participant data falling below thresholds, albeit differently, e.g. participants whose data fell below thresholds was considered missing while participants data which was above thresholds was prorated.^1-4^

Figure S-1: Handling of missing data

**Table S-1: Search strategy in OVID Medline R (ALL)**

| **Concept** | **Layer** | **Search terms** |
| --- | --- | --- |
| **ePGHD** | 1. | Mobile Applications/ |
|  | 2. | Smartphone/ |
|  | 3. | (mobile adj2 (device? or app*)).ti,ab. |
|  | 4. | ((smartphone? or smart) adj2 (phone? or device?)).ti,ab. |
|  | 5. | ((remote? or home?) adj3 (monitor* or track*)).ti,ab. |
|  | 6. | (electronic adj4 (diar* or questionnaire?)).ti,ab. |
|  | 7. | (Patient generated or person generated or caregiver generated or peer generated or PGHD).ti,ab. |
|  | 8. | 1 or 2 or 3 or 4 or 5 or 6 or 7 |
| **Treatment response** | 9. | Treatment Outcome/ |
|  | 10. | "Activities of Daily Living"/ |
|  | 11. | "Quality of Life"/ |
|  | 12. | (predict* adj2 (outcome? or response)).ti,ab. |
|  | 13. | ((treatment* or drug* or medication?) adj2 (effectiveness or efficacy or response*)).ti,ab. |
|  | 14. | (treatment adj3 (outcome* or effect?)).ti,ab. |
|  | 15. | (symptom* adj2 (improve* or response)).ti,ab. |
|  | 16. | ("activities of daily living" or "quality of life" or QoL or early detection or patient reported outcome).ti,ab. |
|  | 17. | (responder? or Non responder?).ti,ab. |
|  | 18. | 9 or 10 or 11 or 12 or 13 or 14 or 15 or 16 or 17 |
|  | 19. | 8 and 18 |
| **Study type** | 20. | limit 19 to "review articles" |
|  | 21. | 19 not 20 |
|  | 22. | (comment or editorial or letter).pt. |
|  | 23. | 21 not 22 |
| **Other** | 24. | 23 not (Animals/ not (Animals/ and Humans/)) |
|  | 25. | limit 24 to english language |
|  | 26. | limit 25 to yr="2018 -Current" |

***Table S-2: Ovid Embase search***

| Concept | Layer | Search terms |
| --- | --- | --- |
| ePGHD | 1. | exp mobile application/ |
|  | 2. | smartphone/ |
|  | 3. | (mobile adj2 (device? or app*)).ti,ab. |
|  | 4. | ((smartphone? or smart) adj2 (phone? or device?)).ti,ab. |
|  | 5. | ((remote? or home?) adj3 (monitor* or track*)).ti,ab. |
|  | 6. | (electronic adj4 (diar* or questionnaire?)).ti,ab. |
|  | 7. | (Patient generated or person generated or caregiver generated or peer generated or PGHD).ti,ab. |
|  | 8. | 1 or 2 or 3 or 4 or 5 or 6 or 7 |
| Treatment response | 9. | treatment outcome/ |
|  | 10. | patient-reported outcome/ |
|  | 11. | daily life activity/ |
|  | 12. | "quality of life"/ |
|  | 13. | (predict* adj2 (outcome? or response)).ti,ab. |
|  | 14. | ((treatment* or drug* or medication?) adj2 (effectiveness or efficacy or response*)).ti,ab. |
|  | 15. | (treatment adj3 (outcome* or effect?)).ti,ab. |
|  | 16. | (symptom* adj2 (improve* or response)).ti,ab. |
|  | 17. | (daily life activity or "quality of life" or QoL or early detection or patient reported outcome).ti,ab. |
|  | 18. | (responder? or Non-responder?).ti,ab. |
|  | 19. | 9 or 10 or 11 or 12 or 13 or 14 or 15 or 16 or 17 or 18 |
|  | 20. | 8 and 19 |
| Source type | 21. | limit 20 to "remove medline records" |
| Publication type | 22. | (comment or editorial or letter).pt. |
|  | 23. | 21 not 22 |
|  | 24. | limit 23 to conference abstract status |
| Other | 25. | 23 not 24 |
|  | 26. | 25 not (Animals/ not (Animals/ and Humans/)) |
|  | 27. | limit 26 to english language |
|  | 28. | limit 27 to yr="2018 -Current" |

Table S-3: Web of Science search (All databases)

| **Concept** | **Layer** | **Search terms** |
| --- | --- | --- |
| **ePGHD** | 1. | ((((((TS=(mobile NEAR/2 (device OR app*) )) OR TS=(smartphone)) OR TS=(smart NEAR/2 (phone? or device?))) OR TS=(remote NEAR/3 (monitor* or track*))) OR TS=(home NEAR/3 (monitor* or track*))) OR TS=(electronic NEAR/4 (diar* or questionnaire?))) OR TS=(patient-generated or caregiver-generated or peer-generated or PGHD) and Preprint Citation Index (Exclude – Database) |
| **Treatment response** | 2. | (((((TS=(predict* NEAR/2 (outcome? or response))) OR TS=((treatment* or drug or medication?*) NEAR/2 (effectiveness or efficacy or response*))) OR TS=(treatment NEAR/3 (outcome* or effect?))) OR TS=(symptom* NEAR/2 (improve* or response*))) OR TS=("activities of daily living" or daily life activity or "quality of life" or QoL or early detection OR patient-reported outcome) OR TS=(responder? or non-responder?) and Preprint Citation Index (Exclude – Database) |
|  | 3. | #1 AND #2 and Preprint Citation Index (Exclude – Database) |
|  | 4. | #1 AND #2 and Preprint Citation Index (Exclude – Database) and MEDLINE® (Exclude – Database) |
|  | 5. | #1 AND #2 and Preprint Citation Index (Exclude – Database) and MEDLINE® (Exclude – Database) and Web of Science Core Collection (Database) |
|  |  | #1 AND #2 and Preprint Citation Index (Exclude – Database) and MEDLINE® (Exclude – Database) and Web of Science Core Collection (Database) and Review Article or Book or Editorial Material or Letter or News (Exclude – Document Types) |
|  |  | #1 AND #2 and Preprint Citation Index (Exclude – Database) and MEDLINE® (Exclude – Database) and Web of Science Core Collection (Database) and Review Article or Book or Editorial Material or Letter or News (Exclude – Document Types) and Animalia or Apis Mellifera or Calyptorhynchus Banksii Graptogyne or Calyptorhynchus Lathami Halmaturinus or Cerambycidae or Danio Rerio or Dendroctonus Brevicomis or Epinephelus Coioides or Hypnomonas or Karenia Mikimotoi or Martes Pennanti or Myotis or Oncorhynchus Mykiss or Physokermes Inopinatus or Salmo Salar or Schistocerca Gregaria or Scolytinae (Exclude – Organisms) |
|  | 18. | #1 AND #2 and Preprint Citation Index (Exclude – Database) and MEDLINE® (Exclude – Database) and Web of Science Core Collection (Database) and Meeting or Review Article or Book or Editorial Material or Letter or News (Exclude – Document Types) and Animalia or Apis Mellifera or Calyptorhynchus Banksii Graptogyne or Calyptorhynchus Lathami Halmaturinus or Cerambycidae or Danio Rerio or Dendroctonus Brevicomis or Epinephelus Coioides or Hypnomonas or Karenia Mikimotoi or Martes Pennanti or Myotis or Oncorhynchus Mykiss or Physokermes Inopinatus or Salmo Salar or Schistocerca Gregaria or Scolytinae (Exclude – Organisms) and English (Languages) |
|  | 19. | #1 AND #2 and Preprint Citation Index (Exclude – Database) and MEDLINE® (Exclude – Database) and Web of Science Core Collection (Database) and Meeting or Review Article or Book or Editorial Material or Letter or News (Exclude – Document Types) and Animalia or Apis Mellifera or Calyptorhynchus Banksii Graptogyne or Calyptorhynchus Lathami Halmaturinus or Cerambycidae or Danio Rerio or Dendroctonus Brevicomis or Epinephelus Coioides or Hypnomonas or Karenia Mikimotoi or Martes Pennanti or Myotis or Oncorhynchus Mykiss or Physokermes Inopinatus or Salmo Salar or Schistocerca Gregaria or Scolytinae (Exclude – Organisms) and English (Languages) and 2024 or 2023 or 2022 or 2021 or 2020 or 2019 or 2018 (Publication Years) |

Table S-4: ACM digital library search

|  |  | “mobile device” OR “mobile devices” OR “mobile app” OR “mobile apps” OR “mobile application” OR “mobile applications” OR smartphone* OR "smart phone" OR “smart phones” OR “smart device” OR “smart devices” OR "remote monitor” or “remote monitoring” OR “remote-monitoring” OR “remote tracking” OR “tracking remotely” OR “home monitor” OR “home-monitor” OR “home monitoring” OR “home-monitoring” OR “home tracking” OR “tracking at home” OR “electronic diary” OR “electronic questionnaire” OR “patient generated” OR “patient-generated” OR “person generated” OR “person-generated” OR “caregiver generated” OR “care-giver generated” OR “caregiver-generated” OR “peer generated” OR “peer-generated” OR PGHD |
| --- | --- | --- |
|  |  | "response prediction" OR "prediction of response" OR "prediction of outcomes" OR "outcome prediction" OR "treatment effectiveness" OR “effectiveness of treatment” OR "treatment efficacy" OR “efficacy of treatment” OR "treatment response" OR “response to treatment” OR "drug effectiveness" OR “effectiveness of a drug” OR "drug efficacy" OR “efficacy of a drug” OR "drug response" OR “response to a drug” OR "medication effectiveness" OR “effectiveness of medication” OR "medication efficacy" OR “efficacy of medication” OR "response to medication" OR "treatment outcome" OR “treatment outcomes” OR “treatment effect” OR “treatment effects” OR "symptom improvement" OR "symptomatic improvement" OR "symptomatic response" OR "activities of daily living" OR “daily life activity” OR "quality of life" OR "early detection" OR "patient reported outcome" OR “patient-reported outcome” OR responder OR "non responder" OR “non-responder” |

Table S-5: Eligibility criteria

|  | Definition | **Examples of inclusion** | **Examples of exclusion** |
| --- | --- | --- | --- |
| **Population** | People of any age with any health condition undergoing any type of treatment  A health condition could be a short or long-term health issue requiring treatment, e.g. lower back pain, obesity.  Treatment should have details of when/how administration happened.  Treatment could include drug therapy, surgery, physical therapy, psychological therapy, lifestyle interventions or treatment delivered through applications. | Studies in which parents completed sleep distress scores on behalf of their children to measure effect of cognitive behavioural therapy delivered via an app  -Studies in which patients underwent end of life care or palliative care e.g., patients with terminal cancer palliatively treated for pain   -Studies in which obese participants were prescribed lifestyle intervention (daily physical exercise and nutrition) delivered through an app.   -Studies in which analgesia was prescribed for pain but that did not specify the health condition causing pain. | Studies evaluating preventative interventions in people without a diagnosed health condition, such as personalized messages that recommended lifestyle changes or doctors’ visits   Studies in which healthy individuals submitted weekly lifestyle summaries (exercise, nutrition) with the goal to monitor and promote healthier lifestyles.  -Studies that predicted clinical outcomes of a disease class (e.g. cardiovascular diseases) without describing treatments administered for the diseases |
| **Concept** | i. High frequency ePGHD   ePGHD included any data relevant for assessing or managing health and disease collected by patients themselves or their family members or caregivers. For example, data on symptoms, biometrics, mental health status, behavioural, lifestyle, adverse events, quality of life, etc.  We defined high frequency as ePGHD completion requested at least once weekly.  ePGHD included data in any format (numeric, text, photographic, video, etc.) collected in digital form and submitted electronically via email, smart devices, weblink, patient portal, text message prompts, web application, etc. | -Studies in which study participants were asked to report impact of drug therapy on ability to work via an electronic diary once a week.  -Studies in which participants were asked to capture images of their post-surgical wounds and answer a series of questions related to their symptoms and recovery every day via a patient portal.  -Studies that evaluated the effect of treatment by asking participants to report sleep quality, sleep duration, and difficulty initiating sleep daily via a smartphone app.  -Studies in which diabetic participants had continuous blood glucose monitors that produced daily summaries to guide coaching advice for blood glucose control via an app. -Studies in which ePGHD was collected continuously but analysed as a daily summary -Studies in which patients submitted blood pressure levels daily via text message with submitted data populating their electronic health record and used to determine treatment response  -Studies in which participants filled in a structured questionnaire regarding their epileptic symptoms weekly via email. | -Studies in which participants with Parkinson’s disease wore body sensors that continuously measured physical activity for analysis of treatment response  -Studies in which healthcare professionals recorded patient symptoms during a weekly video or telephone interview.  - Studies in which patients recorded their data on paper and this was later transcribed into a digital format |
|  | ii. Treatment response-   Our definition of treatment response includes evaluation of treatment efficacy, (comparative) effectiveness, and adverse events following treatment as a primary, secondary or tertiary outcome, or change or change in treatment  We will also include studies that predict/detect (early or lack of) response to treatment, or identify clusters based on types of response | - Studies that assessed the efficacy of an app in reducing symptom burden of chemotherapy adverse effects, such as pain, nausea, vomiting, diarrhoea, infection, tiredness.   -Studies that compared effectiveness of drugs in rheumatoid arthritis using daily symptom data.  -Studies that predicted response to drug treatment in people with Parkinson’s by analysing daily data on keyboard dynamics.  -Studies that used clustered people who experienced pain into groups based on the degree of fluctuation in their pain scores recorded daily in an app following drug treatment ] | -Studies in patients with cystic fibrosis reported their symptoms weekly or sooner to detect exacerbations but without this being linked to treatment.  --Studies that described ePGHD completion rates but without analysing the ePGHD itself in relation to treatment. |
| **Context** | Data collection outside traditional clinic or research settings | Studies in which participants collected symptom data at home via a smartphone or via an online form on a personal computer.  -Studies in which data collection was mixed, e.g., at home and in the waiting room, provided ePGHD is actively reported at least weekly. | -Studies in which participants generated data only in clinic settings with or without the help of healthcare professionals e.g., patients generating physiological data using a wearable device or filling in a symptom questionnaire during hospital admission  -Studies in which participants generated health data within a care home/hospice setting (e.g., from smart sensors to detect falls) |
| **Study type** | Any longitudinal study | Randomised controlled trials, non-randomised comparative effective studies, interventional studies without a control group, observational studies | Cross-sectional studies |

Table S-6: Data extraction items

| **Objective** | **Extracted items** |
| --- | --- |
| 1. Identify and characterise studies that used high frequency time-series ePGHD to understand treatment response | Study title, first author, year of publication, publication type (study protocol, journal article, or conference paper), country study was conducted, study design (randomized controlled trials, observational studies, other interventional studies), health condition being studied, treatment, age group, and number of participants |
| 1. Describe what ePGHD types were collected and how, including ePGHD-based definitions of response | Type of ePGHD, reporter of ePGHD (categorized into patient, proxy, patient and/or proxy), type of reporting (active, passive or both), method of reporting (mobile apps, webforms, connected devices, wearables/biosensors), frequency of reporting (daily or few times a day, once weekly or few times a week, and mix of frequencies), duration of reporting, and ePGHD definition of treatment response.  A proxy could have been a parent or carer reporting on behalf of a patient. Active reporting of ePGHD involves information that patients intentionally record or submit, such as on smartphones, requiring conscious effort and engagement. Passive reporting, in contrast, refers to data collected automatically without direct patient input, typically through sensors or wearables.^5^ We considered treatment response to be defined if a study explicitly gave a definition. Only definitions based on ePGHD were included, meaning the treatment response had to be directly linked to the type of ePGHD reported by participants. For instance, if participants submitted daily blood pressure readings, the treatment response needed to be defined using that data, such as whether participants remained below the clinical threshold for high blood pressure (i.e., 140/90 mmHg). Similarly, if patients tracked daily migraine occurrences and the study defined treatment response as, for instance, a 50% reduction in monthly migraine days, we accepted this as an ePGHD-based definition |
| 1. Describe ePGHD preprocessing and analysis methods | Method(s) applied, objectives of all methods, whether one of the methods accounted for correlation between repeated measures, and if so, which method, the objective of the method that accounted for correlations, how exactly the study accounted for correlations, whether the study has missing data, the level of missingness, whether a study applied a threshold of missingness for participants to be included in analysis, and if present, what the threshold was, handling of participants whose data fell above and below this threshold, whether the impact of missing data was determined using a sensitivity analysis and the methods used in the such analysis, study outcomes (primary, secondary, tertiary, etc), whether the study was powered to analyse any ePGHD outcome, ePGHD processing before analysis (single summaries over time, multiple summaries, specific time points/periods, every data point), and reporting pattern. We decided to extract reporting patterns because they can impact treatment response estimates. In longitudinal studies, declining reporting over time is common and can introduce bias if participants who continue reporting differ systematically, for example, if those who are more unwell or, conversely, those who are healthier are more likely to continue reporting. Although our objective was not to assess the extent of potential bias, we were interested in evaluating the transparency with which studies detailed reported patterns. We categorized ePGHD reporting patterns as ‘complete’ when all participants provided data as expected; ‘declining’ when the study reported a reduction in reporting over time; and ‘incomplete but sustained’ when some data were missing but a consistent number of participants reported throughout the study period.  Some studies used more than one analytical method or data preprocessing approach. In these cases, we counted only the method or approach that made the fullest use of available data. For preprocessing, approaches were ranked D > B > C > A, with D considered the most comprehensive and A the least. For example, if a study used both approaches A and D, only D was recorded. We considered approach B preferable to C because it did not exclude any data from analysis. When tallying analytical methods, we applied the same principle within each study objective (treatment response estimation, prediction, or clustering): only the most rigorous method was counted. For example, if both longitudinal and cross-sectional methods were applied, only the longitudinal method was recorded. Within these groupings, each distinct method was counted every time it appeared. Descriptive statistics were only counted if they were used alone—for example, when a study simply reported how many participants crossed a treatment-response threshold at a specific time point without complementing these with inferential methods. If a study used descriptive statistics alongside inferential analyses, only the inferential method was counted. Despite their ability to only consider two data points, we counted paired t-tests under longitudinal measures because they account for correlations between repeated measures. |

**Preferred Reporting Items for Systematic reviews and Meta-Analyses extension for Scoping Reviews (PRISMA-ScR) Checklist**

| **SECTION** | **ITEM** | **PRISMA-ScR CHECKLIST ITEM** | **REPORTED ON PAGE #** |
| --- | --- | --- | --- |
| **TITLE** | | | |
| Title | 1 | Identify the report as a scoping review. | 1 |
| **ABSTRACT** | | | |
| Structured summary | 2 | Provide a structured summary that includes (as applicable): background, objectives, eligibility criteria, sources of evidence, charting methods, results, and conclusions that relate to the review questions and objectives. | 2 |
| **INTRODUCTION** | | | |
| Rationale | 3 | Describe the rationale for the review in the context of what is already known. Explain why the review questions/objectives lend themselves to a scoping review approach. | 3 |
| Objectives | 4 | Provide an explicit statement of the questions and objectives being addressed with reference to their key elements (e.g., population or participants, concepts, and context) or other relevant key elements used to conceptualize the review questions and/or objectives. | 3 |
| **METHODS** | | | |
| Protocol and registration | 5 | Indicate whether a review protocol exists; state if and where it can be accessed (e.g., a Web address); and if available, provide registration information, including the registration number. | 14 |
| Eligibility criteria | 6 | Specify characteristics of the sources of evidence used as eligibility criteria (e.g., years considered, language, and publication status), and provide a rationale. | 14/ 6-8 in supplementary file |
| Information sources* | 7 | Describe all information sources in the search (e.g., databases with dates of coverage and contact with authors to identify additional sources), as well as the date the most recent search was executed. | 4,14 |
| Search | 8 | Present the full electronic search strategy for at least 1 database, including any limits used, such that it could be repeated. | 3-5 in supplementary file |
| Selection of sources of evidence† | 9 | State the process for selecting sources of evidence (i.e., screening and eligibility) included in the scoping review. | 4, 15 |
| Data charting process‡ | 10 | Describe the methods of charting data from the included sources of evidence (e.g., calibrated forms or forms that have been tested by the team before their use, and whether data charting was done independently or in duplicate) and any processes for obtaining and confirming data from investigators. | 15 |
| Data items | 11 | List and define all variables for which data were sought and any assumptions and simplifications made. | 15/ 10 in supplementary file |
| Critical appraisal of individual sources of evidence§ | 12 | If done, provide a rationale for conducting a critical appraisal of included sources of evidence; describe the methods used and how this information was used in any data synthesis (if appropriate). | NA |
| Synthesis of results | 13 | Describe the methods of handling and summarizing the data that were charted. | 15, 9-10 in supplementary file |
| **RESULTS** | | | |
| Selection of sources of evidence | 14 | Give numbers of sources of evidence screened, assessed for eligibility, and included in the review, with reasons for exclusions at each stage, ideally using a flow diagram. | 4 |
| Characteristics of sources of evidence | 15 | For each source of evidence, present characteristics for which data were charted and provide the citations. | Supplementary file 2 |
| Critical appraisal within sources of evidence | 16 | If done, present data on critical appraisal of included sources of evidence (see item 12). | N/A |
| Results of individual sources of evidence | 17 | For each included source of evidence, present the relevant data that were charted that relate to the review questions and objectives. | Supplementary file 2 |
| Synthesis of results | 18 | Summarize and/or present the charting results as they relate to the review questions and objectives. | 3-11 |
| **DISCUSSION** | | | |
| Summary of evidence | 19 | Summarize the main results (including an overview of concepts, themes, and types of evidence available), link to the review questions and objectives, and consider the relevance to key groups. | 11-12 |
| Limitations | 20 | Discuss the limitations of the scoping review process. | 12 |
| Conclusions | 21 | Provide a general interpretation of the results with respect to the review questions and objectives, as well as potential implications and/or next steps. | 12-13 |
| **FUNDING** | | | |
| Funding | 22 | Describe sources of funding for the included sources of evidence, as well as sources of funding for the scoping review. Describe the role of the funders of the scoping review. | 15 |

Bibliography

1. Becker WJ, Spacey S, Leroux E, Giammarco R, Gladstone J, Christie S, et al. A real-world, observational study of erenumab for migraine prevention in Canadian patients. Headache. 2022;62(4):522-9.

2. Goadsby PJ, Friedman DI, Holle-Lee D, Demarquay G, Ashina S, Sakai F, et al. Efficacy of Atogepant in Chronic Migraine With and Without Acute Medication Overuse in the Randomized, Double-Blind, Phase 3 PROGRESS Trial. Neurology. 2024;103(2):e209584.

3. Lipton RB, Gandhi P, Tassorelli C, Reuter U, Harriott AM, Holle-Lee D, et al. Early Improvements With Atogepant for the Preventive Treatment of Migraine: Results From 3 Randomized Phase 3 Trials. Neurology. 2025;104(2):e210212.

4. Tassorelli C, Jensen RH, Goadsby PJ, Charles AC, Tepper SJ, Snoer AH, et al. Long-term safety, tolerability, and efficacy of eptinezumab in chronic cluster headache (CHRONICLE): an open-label safety trial. Lancet Neurol. 2025;24(5):429-40.

5. Bourke A, Dixon WG, Roddam A, Lin KJ, Hall GC, Curtis JR, et al. Incorporating patient generated health data into pharmacoepidemiological research. Pharmacoepidemiology and Drug Safety. 2020;29(12):1540-9.
